# Food-based formula modulates gut microbial homeostasis and inflammatory responses following LPS challenge in mice

**DOI:** 10.3389/fnut.2026.1932946

**Published:** 2026-10-01

**Authors:** Xiaolan Ying, Jing Li, Yue Tang, Guoliang Lu, Li Hong, Zhiyan Zhan

**Affiliations:** 1 Department of Clinical Nutrition, Shanghai Children's Medical Center, Shanghai Jiao Tong University School of Medicine, Shanghai, China; 2 Department of Respiratory Medicine, Shanghai Children's Medical Center, Shanghai Jiao Tong University School of Medicine, Shanghai, China; 3 Department of Pediatrics, Affiliated Hospital of Guizhou Medical University, Guiyang, Guizhou, China; 4 Clinical Research Center, Shanghai Children’s Medical Center, Shanghai Jiao Tong University School of Medicine, Shanghai, China

**Keywords:** dietary intervention, food-based formula, gut microbiota, inflammation, intestinal barrier, oral nutritional supplement

## Abstract

**Background:**

Oral nutritional supplements (ONSs) are widely used to support patients with malnutrition or chronic diseases. However, their potential effects on inflammatory recovery and gut microbial composition remain insufficiently understood. This study evaluated a novel whole-food-based formula (FBF) composed of natural food-derived ingredients and investigated its effects on inflammation recovery and gut microbiota in a murine model of lipopolysaccharide (LPS)-induced low-grade inflammation.

**Methods:**

21 male C57BL/6J mice were randomly assigned to a healthy control (HC) group, a milk-based formula (MBF) group, or a food-based formula (FBF) group. Mild systemic inflammation was induced by intraperitoneal injection of LPS (3 mg/kg). After LPS administration, mice received dietary intervention for 10 days. Serum inflammatory cytokines, colonic histopathology, and gut microbiota profiles were evaluated by ELISA, hematoxylin–eosin staining, and 16S rRNA gene sequencing, respectively.

**Results:**

Serum IL-6 concentrations were elevated in the MBF group compared with healthy controls (*p* < 0.05), whereas no significant difference was observed between the FBF group and healthy controls. Histological evaluation showed a tendency toward reduced mucosal disruption and inflammatory cell infiltration in FBF-treated mice, although the differences in histological scores were not statistically significant. FBF was also associated with increased microbial richness and diversity, as indicated by higher ACE, Chao1, Shannon, and Simpson indices. Principal coordinate analysis and non-metric multidimensional scaling revealed a microbial community structure closer to that of healthy controls in the FBF group. Taxonomic analysis revealed that FBF reduced the relative abundance of *Erysipelotrichaceae* and maintained *Lachnospiraceae_NK4A136_group* abundance.

**Conclusion:**

A whole-food-based nutritional formula was associated with lower serum IL-6 levels and distinct gut microbial compositional profiles compared with a milk-based formula following LPS-induced inflammation. These findings suggest FBF may influence inflammatory recovery and gut microbial composition; however, further functional and mechanistic studies are required to clarify its biological effects.

## 1 Introduction

Chronic low-grade inflammation is increasingly recognized as a common pathological process underlying a wide spectrum of diseases, including obesity, cardiovascular disease, diabetes, chronic kidney disease, and cancer (1, 2). Persistent inflammatory activation not only accelerates disease progression but also impairs tissue recovery and contributes to unfavorable clinical outcomes (3). Consequently, nutritional strategies capable of simultaneously improving nutritional status and modulating inflammatory responses have attracted growing interest (4).

Oral nutritional supplements (ONSs) are commonly prescribed to patients who are unable to meet nutritional requirements through habitual dietary intake (5, 6). They are widely applicable to patients requiring nutritional support, including those undergoing major surgery, receiving anti-tumor therapy, or suffering from frailty or sarcopenia. Accumulating studies have suggested that ONSs can improve nutritional status, reduce complications, and enhance recovery (7–9). However, most commercially available formulas are primarily formulated to provide energy and essential nutrients, while their potential effects on inflammation and host-microbiota interactions remain incompletely understood (10, 11). A previous study indicated that one type of ONSs could improve nutritional status, enhance antioxidant capacity, and mitigate inflammation in dialysis patients (12). Another formulation, particularly when combined with probiotics, may exert potential ant-inflammation effect in malnourished hemodialysis patients (13). Given the close association between malnutrition and chronic inflammation, the development of nutritional formulations with additional biological functions may offer potential clinical benefits.

Dairy matrices are increasingly recognized as functional foods containing bioactive components capable of modulating host physiology beyond their nutritional value (14, 15). However, the mechanisms linking dairy-derived components to intestinal microbiota and inflammatory recovery remain incompletely understood (14, 15). Accumulating evidence indicates that the gut microbiota plays an important role in regulating host metabolism (16), immunity (17), and intestinal barrier function (18). Dietary composition is one of the major determinants of gut microbial structure and function (19, 20). Recent studies suggest that dairy-derived matrices may influence intestinal inflammation through microbiota remodeling and restoration of epithelial barrier function (14). In addition, dietary fibers, plant-derived bioactive compounds, and natural bioactive polysaccharides (21) promote microbial diversity and increase the production of short-chain fatty acids (SCFAs) (22), which contribute to intestinal epithelial integrity and immune homeostasis (23–26).

Recently, increasing attention has been directed toward nutritional formulas containing food-derived ingredients (27). Unlike conventional milk-based formulas, food-based formulas contain diverse substrates derived from whole foods (28), including grains, fruits, vegetables, and fermentable fibers. These components provide diverse nutritional and bioactive compounds that may shape the intestinal ecosystem through microbiota-metabolite interactions. Nevertheless, the effects of food-based formulas on gut microbiota modulation and post-inflammatory recovery remain largely unexplored.

Therefore, we developed a novel whole-food-based formula enriched with natural food-derived ingredients and prebiotic components. Using a mouse model of lipopolysaccharide (LPS)-induced low-grade inflammation, we aimed to evaluate whether this formula could promote inflammation recovery through modulation of gut microbial homeostasis, with a focus on systemic inflammatory responses, intestinal mucosal integrity, and gut microbial composition. We hypothesized that FBF supplementation would be associated with improved inflammatory recovery and modulation of gut microbial homeostasis.

## 2 Materials and methods

### 2.1 Animal model and dietary intervention

21 four-week-old male C57BL/6J mice were obtained from a commercial supplier (Certificate No. SCXK (Shanghai) 2022–0011). All mice were housed under specific pathogen-free (SPF) conditions at a constant temperature (20–24 °C), humidity (45–60%), and a 12 h light/dark cycle. After a 7-day acclimation period, mice were randomly assigned into three groups (*n* = 7 per group): healthy control (HC) group, milk-based formula (MBF) group, and food-based formula (FBF) group. Mice were housed in two cages per group, with 3–4 mice per cage. The experimental protocol was approved by the Animal Ethics Committee of Shanghai Yishang Biotechnology Co., Ltd. (approval No. IACUC-2025-NI-082). A schematic overview of the study design is presented in Figure 1A. Mild systemic inflammation was induced by intraperitoneal injection of LPS (3 mg/kg body weight). Mice in the HC group received an equal volume of sterile 0.9% saline. LPS was prepared in sterile saline at a final concentration of 200 μg/mL. Dietary intervention was initiated following LPS administration and lasted for 10 consecutive days. Body weight was recorded daily throughout the experimental period.

**Figure 1:**
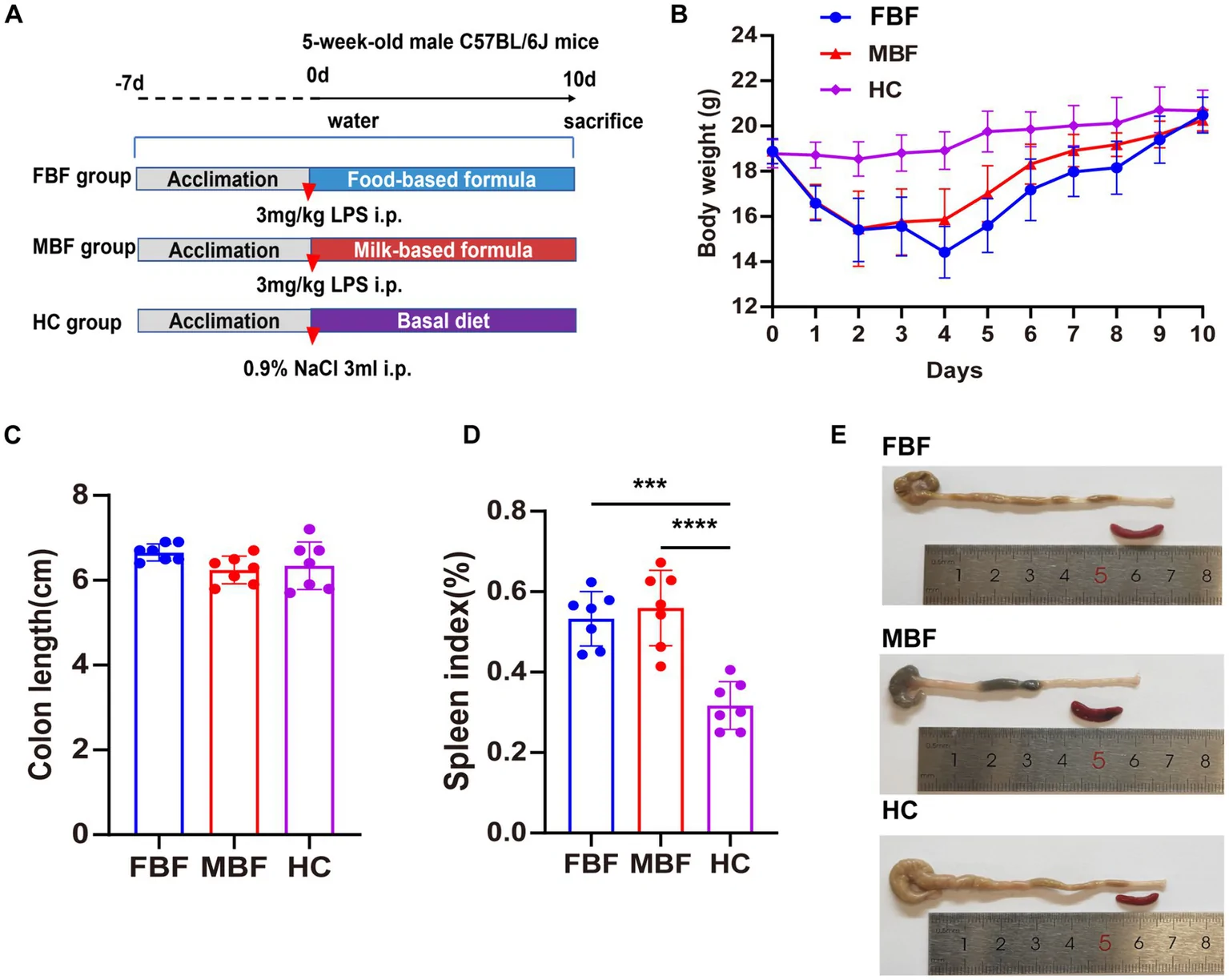
Effects of different dietary interventions on general physiological indicators in LPS-challenged mice. **(A)** experimental protocol, **(B)** body weight, **(C)** colon length, **(D)** spleen index, **(E)** representative images of the mouse colon and spleen in Day 10. Data are represented as mean ± SD. *n* = 7, ^***^p < 0.001, ^****^p < 0.0001.

### 2.2 Formula composition and administration

The nutritional composition and physicochemical characteristics of the two experimental formulas are summarized in Table 1. A commercially available milk-based formula (Nuiren JUNIOR, Nestlé, Switzerland) was used as the formula control (MBF). The food-based formula (FBF) was developed using natural food-derived ingredients including grains, fruits, vegetables, and other nutritional and prebiotic components. Detailed ingredient composition is provided in Supplementary Table S1.

**Table 1:** Nutritional composition (%) and physicochemical properties of the two formulas.

Parameters	Milk-based formula (MBF)	Food-based formula (FBF)
Nutritional components		
Crude protein	13.9%	12.0%
Crude fat	18.3%	30.0%
Carbohydrate	60%	58%
Calcium	0.37%	0.41%
Crude fiber	0.8%	6.0%
Total phosphorus	0.25%	0.28%
Physicochemical properties		
Energy (kilojoule/100 g)	1940	1882
Osmotic pressure (mOsmol/kg)	387	360

The fruit and vegetable components were prepared using an endogenous pectin gel microparticle technology involving mild blanching (85–90 °C, 10–20s), high-pressure microfluidization (120–160 MPa, 3–5 cycles, ≤30 °C), pectin de-esterification, calcium-mediated crosslinking, and low-temperature freeze-drying. All components were subsequently mixed using an air-flow mixer for 5–15 min to obtain the final FBF powder.

During the intervention period, mice received FBF or MBF solution ad libitum as a dietary replacement for the basal diet. The formula powder was dissolved double-distilled water (8 g /30 mL) and provided in drinking bottles. Fresh solutions were supplied every 12 h, and regular drinking water was continuously available.

### 2.3 Sample collection

At the end of the experiment, mice were deeply anesthetized with isoflurane. Blood samples were collected via retro-orbital bleeding and centrifuged at 3500 rpm for 5 min at 4 °C to obtain serum. Spleens were harvested, weighed and stored at −80 °C. Approximately 1 cm of distal colon tissue was excised, washed with phosphate-buffered saline (PBS), and fixed in 4% paraformaldehyde (PFA) for histological evaluation. The spleen index was calculated as follows: *Spleen index (%) = (spleen weight/body weight) × 100%*. Fresh fecal samples were collected individually under sterile conditions and stored at −80 °C for subsequent microbiota analysis.

### 2.4 Histological analysis of Colon tissue

Colon tissues were collected and fixed in 4% PFA for 24 h, followed by dehydration, paraffin embedding, sectioning and hematoxylin–eosin (H&E) staining. Stained sections were scanned and analyzed using a Leica Versa 8 system (Leica, Wetzlar, Germany). Histological evaluation was performed in three mice per group. Two representative intestinal sections from each mouse were examined, and three randomly selected fields per section were analyzed. Histopathological evaluation was performed to measure crypt damage (0–4 points), inflammation infiltration (0–3 points), and goblet cell injury (0–3 points) adapted from previously described histopathological scoring systems (29). Detailed scoring criteria were shown in Supplementary Table S2.

### 2.5 Cytokine analysis

Serum levels of proinflammatory cytokines including tumor necrosis factor-α (TNF-α), interleukin-6 (IL-6) and interleukin-1β (IL-1β) were quantified using respective commercial Enzyme-Linked Immunosorbent Assay (ELISA) kits according to the manufacturer’s instructions. All ELISA kits were obtained from Jianglai Biological Technology Co., Ltd. (Shanghai, China).

### 2.6 Fecal gut microbiome analysis

Total genomic DNA was purified from fecal samples using a MagPure Soil DNA KF Kit (Magen, China). DNA quality and concentration were verified with NanoDrop and agarose gel. Amplification of the bacterial 16S rRNA gene was performed using barcoded primers targeting the V3–V4 hypervariable regions (forward primer: 343F, 5′-TACGGRAGGCAGCAG-3′; reverse primer: 798R, 5′-AGGGTATCTAATCCT-3′) with Tks Gflex DNA Polymerase (Takara, Japan). Amplicon quality was visualized using gel electrophoresis, purified with AMPure XP beads (Agencourt, USA), and amplified for another round of PCR amplification. After purified with the AMPure XP beads again, the final amplicon was quantified using Qubit dsDNA assay kit (Life Technologies, Carlsbad, CA, USA). Equal amounts of purified amplicon were pooled for sequencing. Sequencing was performed on an Illumina NovaSeq 6,000 platform using paired-end 250 bp reads (Illumina Inc., San Diego, CA, USA; OE Biotech Co., Ltd., Shanghai, China).

Raw paired-end reads (FASTQ) were processed using the QIIME2 pipeline (version 2020.11). Primer sequences were removed using Cutadapt trim-paired with primers 343F and 798R. The quality filtering, denoising, sequence merging, and chimera removal were performed using DADA2 with parameters of truncLen = 0/0, truncQ = 2, and maxEE = 4 for both forward and reverse reads. Chimeric sequences were removed using the consensus method with minFoldParentOverAbundance = 1. Amplicon sequence variants (ASVs) were inferred using the DADA2 algorithm, and an ASV abundance table was generated for downstream analyses. The number of observed ASVs per sample ranged from 132 to 329. Representative ASV sequences were taxonomically assigned against the Silva database (version 138) using the q2-feature-classifier plugin with default parameters. No negative control samples were included in this study.

### 2.7 Bioinformatics analysis

Gut microbiota bioinformatic analysis was conducted on the OE Cloud platform^1^. Based on ASV profiles, rarefaction analysis was conducted to assess sequencing depth. Alpha diversity was evaluated using Sobs, ACE, Chao1, Shannon, and Simpson indices. Beta diversity was assessed based on Bray–Curtis dissimilarity matrices. Principal component analysis (PCA) and principal coordinate analysis (PCoA) and non-metric multidimensional scaling (NMDS) were performed to visualize differences in microbial community structure among groups. Statistical differences in community composition were evaluated using permutational multivariate analysis of variance (PERMANOVA; adonis2 function in the vegan package) with 999 permutations. Homogeneity of multivariate dispersion was assessed using PERMDISP before interpreting PERMANOVA results. The Spearman’s correlation analysis was performed to assess associations between the relative abundance of the top 20 bacterial taxa (at genus or family level) and cytokine levels. Multiple comparisons were corrected using the false discovery rate (FDR) method.

### 2.8 Statistical analysis

All data are expressed as mean ± standard deviation (SD). One-way analysis of variance (ANOVA) was used for intergroup comparisons, followed by Dunnett’s multiple comparison test where appropriate. Longitudinal body weight changes were analyzed using two-way repeated-measures ANOVA with treatment group and time as factors, followed by *post hoc* analysis for multiple comparisons when appropriate. A two-sided *p* value <0.05 was considered statistically significant. All statistical analyses were performed using GraphPad Prism 10.1.2 (GraphPad Software Inc., La Jolla, CA, USA). Intestinal flora data processing and visualization were conducted on the OE Cloud platform.

## 3 Results

### 3.1 Food-based formula influenced recovery from LPS-induced low-grade inflammation

To evaluate general physiological responses following dietary intervention, body weight changes, organ indices, and inflammatory-related parameters were assessed.

Following LPS administration, mice in both intervention groups experienced transient weight loss, followed by gradual recovery beginning 3–5 days after LPS challenge. In contrast, mice in the HC group maintained continuous weight gain throughout the experiment (Figure 1B). In both FBF and MBF groups, body weight increased progressively over time (time effect, *p* < 0.0001). Although a significant group × time interaction was observed (*p* = 0.0001), no significant differences were detected between FBF and MBF groups at any individual time point after Bonferroni correction. Colon length did not differ significantly among groups (Figure 1C). Representative images of the colon and spleen were presented in Figure 1E. Because splenic enlargement reflects systemic immune activation, spleen indices were further examined. Compared with HC mice, both LPS-treated groups exhibited significantly increased spleen indices (*p* < 0.001; *p* < 0.0001; *n* = 7), indicating the induction of systemic inflammatory activation. No significant difference was observed between the MBF and FBF groups (Figure 1D). These results suggested that the general condition between the two dietary intervention groups were comparable.

Histopathological analysis of colon tissue further evaluated intestinal inflammatory injury. Representative H&E staining showed preserved epithelial structure and intact crypt architecture in the HC group. In contrast, LPS exposure induced mucosal disruption, glandular injury, and inflammatory cell infiltration. Compared with the MBF group, mice receiving FBF displayed improved mucosal organization, reduced inflammatory infiltration, and a trend toward better preservation of crypt morphology (Figure 2A). Although histological scores did not reach statistical significance, the FBF group consistently showed lower pathological scores than the MBF group (Figure 2B).

**Figure 2:**
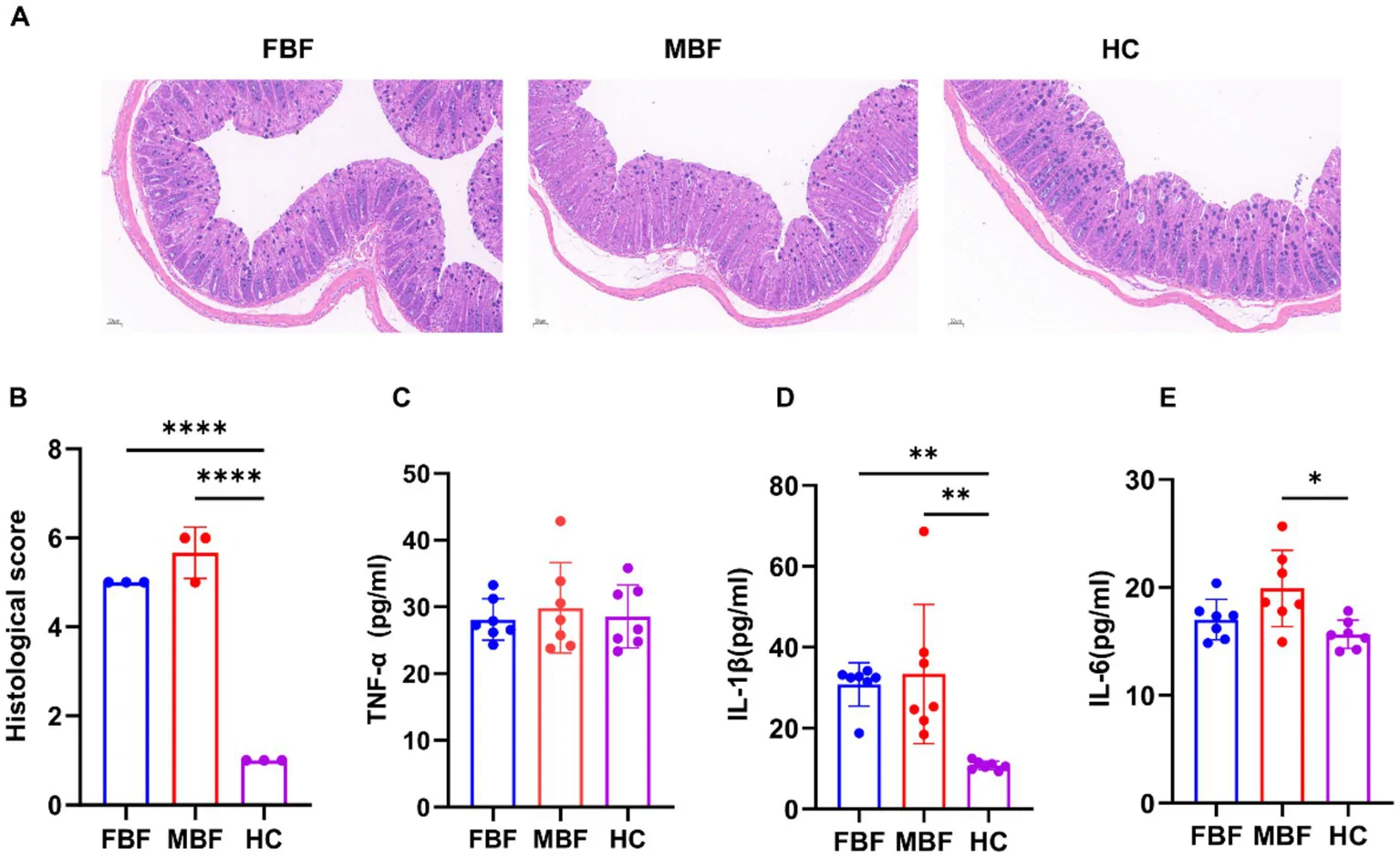
Histopathological changes and serum proinflammatory cytokines in colon tissues. **(A)** HE staining of colon (×100; scale bar 50 μm), **(B)** colon histological score, **(C–E)** serum inflammatory factors TNF-*α*, IL-1β, IL-6. *n* = 7, ^*^p < 0.05, ^**^p < 0.01, ^****^p < 0.0001.

To further characterize the effects of dietary intervention during recovery from LPS-induced inflammation, serum cytokines were quantified. TNF-α levels did not differ significantly among groups (Figure 2C). IL-1β concentrations increased following LPS challenge and remained elevated in both intervention groups relative to HC animals (Figures 2D, *p* < 0.01, *n* = 7). In contrast, serum IL-6 concentrations were elevated in the MBF group compared with HC mice (*p* < 0.05, *n* = 7), whereas IL-6 levels in the FBF group were comparable to those in HC mice (Figure 2E). Collectively, these findings suggest that FBF may facilitate recovery from LPS-induced mild systemic inflammation, as reflected by improved intestinal histological features and recovery of selected inflammatory responses.

### 3.2 Food-based formula improved gut microbial richness and diversity

Given the important role of the intestinal microbiota in immune regulation, 16S rRNA sequencing was performed to investigate whether dietary intervention influenced microbial community characteristics.

Rarefaction analysis indicated sufficient sequencing depth to capture microbial diversity across all samples (Supplementary Figure S1). Alpha diversity analysis revealed marked differences among groups. Compared with HC mice, microbial richness and diversity were reduced following LPS exposure. Specifically, the MBF group showed lower ACE and Chao1 indices, suggesting reduced species richness. Similarly, Shannon and Simpson indices suggested impaired microbial diversity. Mice receiving FBF exhibited consistently increased alpha diversity indices compared with the MBF group (Figures 3A–D), suggesting improved microbial richness and ecological complexity.

**Figure 3:**
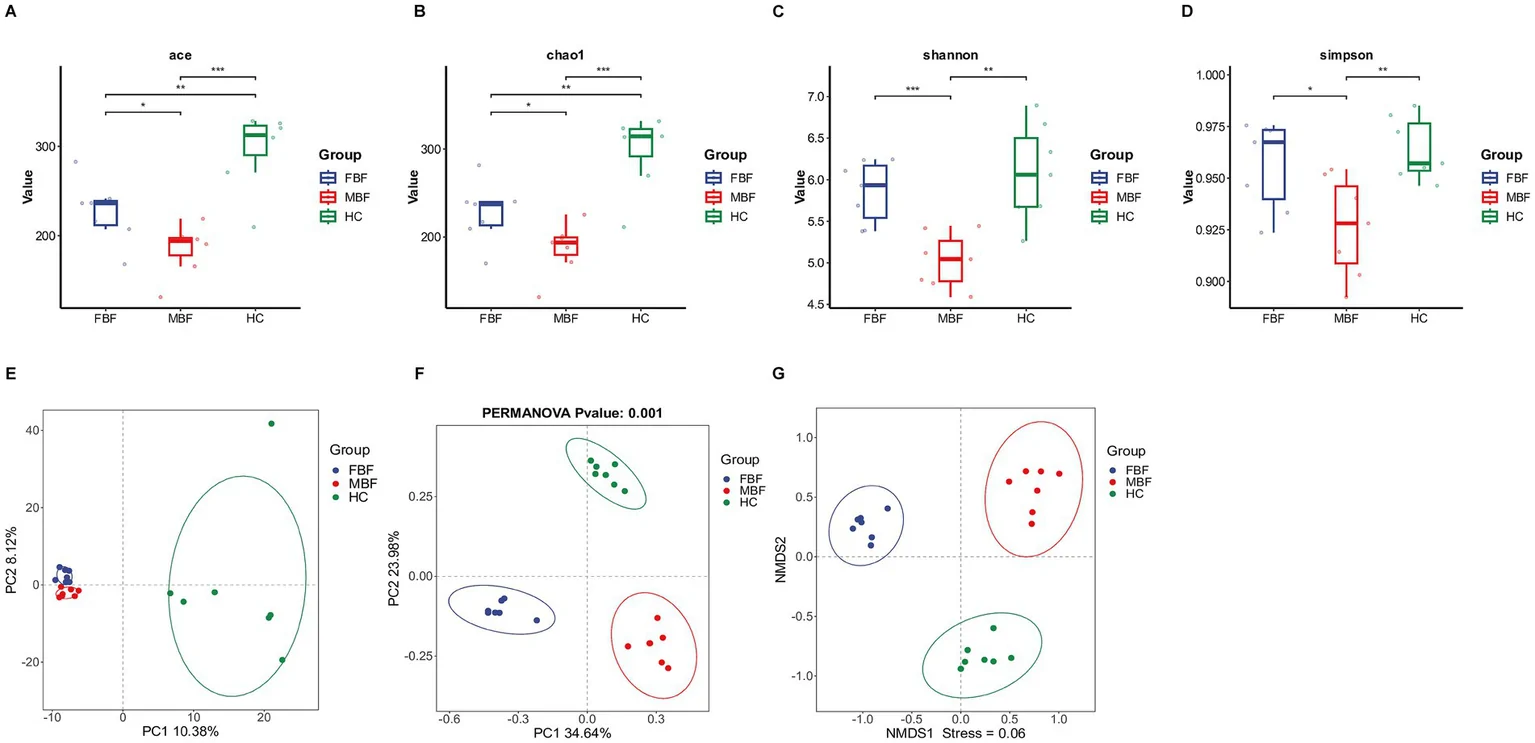
Cluster and diversity analysis of gut microbiota in different groups. **(A–D)** Reflecting the alpha diversity of fecal microbiota, ace, Chao1, Shannon, Simpson in each group. **(E)** PCA plot of gut microbial community. **(F)** PCoA plot based on Bray–Curtis dissimilarity. **(G)** NMDS plot of gut microbial communities. PCA, principal component analysis; PCoA, principal coordinate analysis; NMDS, non-metric Multidimensional Scaling. *n* = 7, ^*^p < 0.05, ^**^p < 0.01, ^***^p < 0.001.

To further evaluate overall microbial structure, beta diversity analyses were performed. Principal component analysis (PCA) demonstrated clear separation between healthy and LPS-treated mice (Figure 3E). Principal coordinate analysis (PCoA) based on Bray-Curtis distance showed clustering patterns among groups (Figure 3F). PERMANOVA revealed significant differences in overall microbial community composition among groups (*f* = 11.892, *R^2^* = 0.569, *p* = 0.001, 999 permutations). PERMDISP analysis showed no significant differences in within-group dispersion (*p* > 0.05), indicating that the observed differences were attributable to shifts in community composition rather than differences in dispersion. Consistently, non-metric multidimensional scaling (NMDS) based on Bray-Curtis distances demonstrated that microbial communities in FBF-treated mice clustered closer to HC animals than those in the MBF group (Figure 3G).

These findings suggest that FBF promoted recovery of LPS-induced dysbiosis by improving microbial diversity and richness and shifting the overall community structure toward that of healthy controls.

### 3.3 Food-based formula modulated gut microbiota composition

To characterize specific microbial alterations associated with dietary intervention, taxonomic composition was analyzed across experimental groups.

At the phylum level, *Firmicutes* and *Bacteroidetes* were the dominant across all groups, and no significant differences were observed in the Firmicutes/Bacteroidetes ratio (Figure 4A). At finer taxonomic resolution, distinct differences emerged. At the family level, relative abundance of *Erysipelotrichaceae* was elevated in the MBF group but remained comparatively lower in FBF-treated mice (Figure 4B). At the genus level, the relative abundance of *Lachnospiraceae_NK4A136_group* was reduced following MBF intervention but remained comparable between FBF and HC mice (Figure 4C). Community distribution plots at family and genus levels further illustrated that microbial composition in the FBF group more closely aligned with those of HC groups (Figures 4D–E).

**Figure 4:**
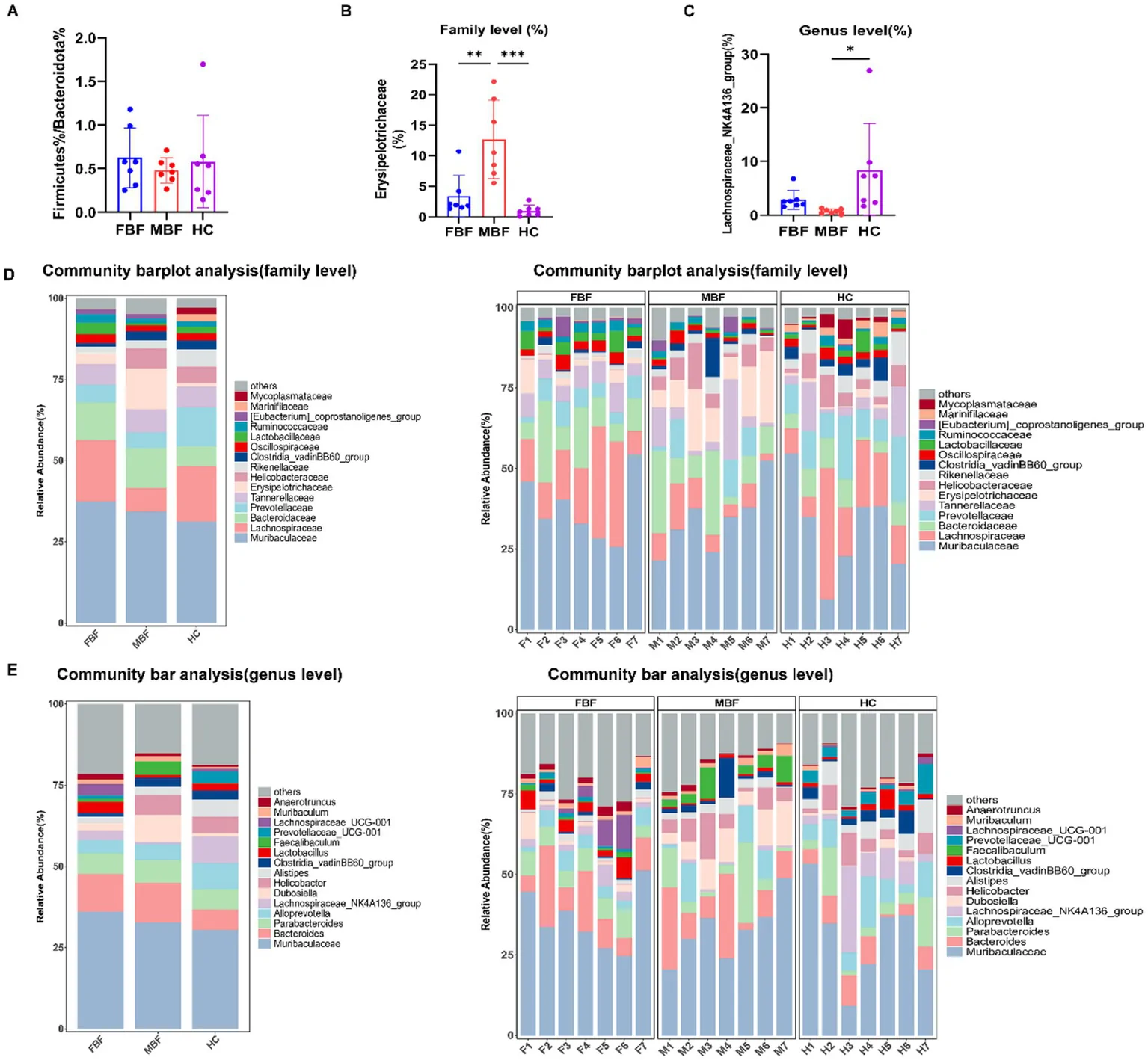
Relative abundance analysis of gut microbiota at different taxonomic levels. **(A)** Firmicutes%/Bacteroidetes% ratio in each group; **(B)** Relative abundances of *Erysipelotrichaceae* (family level); **(C)** Relative abundances of *Lachnospiraceae_NK4A136_group* (genus level); **(D–E)** Relative abundances of the gut microbiota at family and genus levels. Left panel: each column represents one group; Right panel: each column represents one individual sample. *n* = 7, ^*^p < 0.05, ^**^p < 0.01, ^***^p < 0.001.

Together, these results indicate that FBF induced selective remodeling of the intestinal microbiota and promoted a shift toward a microbial profile resembling that of healthy controls following LPS-induced dysbiosis.

### 3.4 Correlation between gut microbial taxa and serum cytokines

To explore potential associations between gut microbial alterations and systemic inflammatory responses, Spearman’s correlation analysis was performed between the relative abundances of selected bacterial taxa and serum cytokine levels.

At the genus level, *Faecalibaculum* was positively correlated with serum IL-1β levels (Figure 5A). In contrast, correlations between individual bacterial genera and TNF-α or IL-6 levels were generally weaker and did not reach statistical significance. The relative abundance of *Lachnospiraceae_NK4A136_group* showed negative correlations with all three inflammatory cytokines, although these associations did not reach statistical significance.

**Figure 5:**
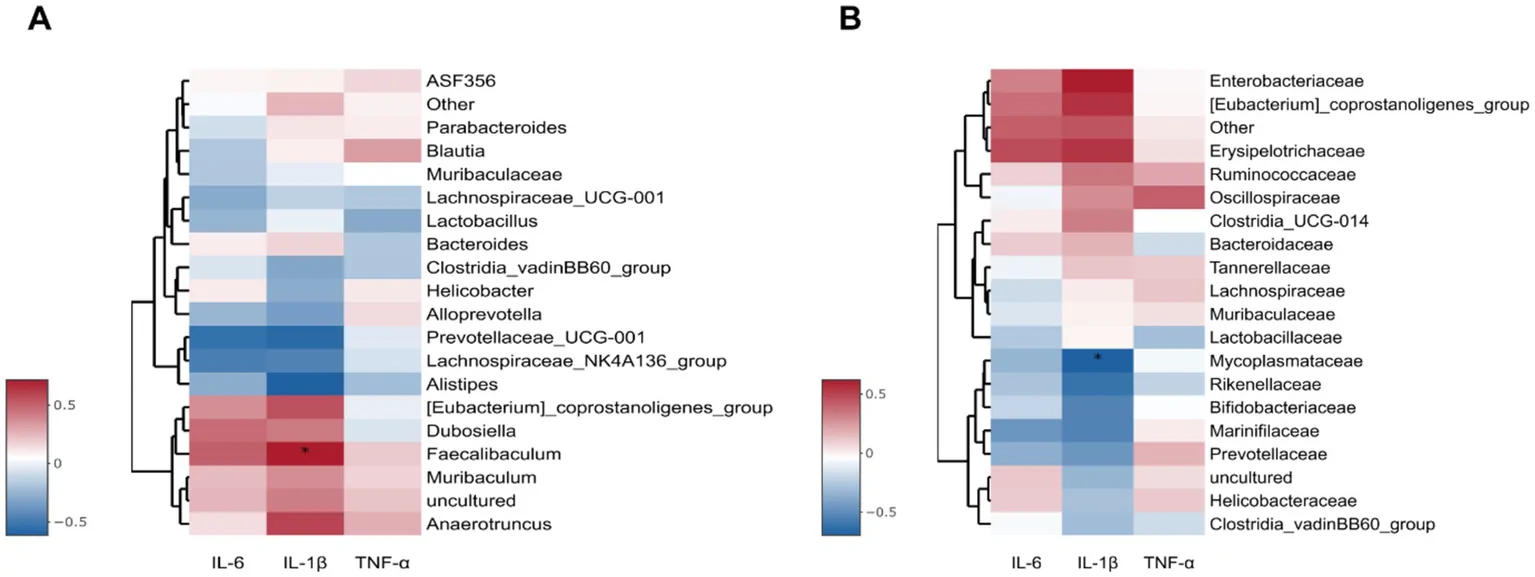
Correlation analysis of intestinal flora. **(A)** genus level; **(B)** family level. *n* = 7, ^*^p < 0.05.

At the family level, *Mycoplasmataceae* exhibited a negative association with IL-1β levels (Figure 5B). *Erysipelotrichaceae* showed positive correlations with IL-1β and IL-6. However, these correlations did not remain statistically significant after multiple-comparison correction.

Overall, these findings suggest that specific microbial taxa, particularly *Faecalibaculum* and *Erysipelotrichaceae*, were associated with inflammatory cytokine profiles, although further studies are required to determine their functional roles in inflammation recovery.

## 4 Discussion

Inflammation is an essential physiological response that maintains host homeostasis in response to pathogenic or damaging stimuli. Low-grade chronic inflammation has been implicated in the initiation and progression of various chronic diseases (1). Although ONSs play an important role in nutritional management of chronic diseases, their specific role in regulating inflammatory responses and gut microbial homeostasis remain insufficiently understood (7). In this study, we developed a whole-food-based formula and evaluated its effects in an LPS-induced low-grade inflammation model. Although LPS-induced inflammatory responses typically peak within 24–48 h (30), subsequent processes including immune homeostasis, intestinal barrier repair, and microbiota reassembly may continue for days thereafter (31). Thus, the 10-day post-LPS period was considered a recovery phase during which dietary intervention could potentially exert its effect. Compared with a conventional milk-based formula, FBF was associated with improved recovery of intestinal histological features, normalization of circulating IL-6 levels toward those observed in healthy controls, increased microbial richness and diversity, and a shift in microbial community structure toward that of healthy animals. These findings support the concept that dietary composition may influence the recovery process following inflammation and highlight the potential role of food-based formulations in modulating host-microbiota interactions.

As a dietary intervention administered following LPS challenge, FBF appeared to selectively influence inflammatory recovery rather than broadly suppress inflammatory mediators. Previous studies have suggested that nutritional interventions administered after inflammatory stimulation may modify subsequent inflammatory responses (32). Among inflammatory mediators evaluated in this study, IL-6 was the only cytokine affected by FBF intervention. IL-6 is a multifunctional cytokine that contributes to inflammatory amplification, intestinal epithelial dysfunction, and sustained immune activation (33, 34). Elevated IL-6 levels have been associated with impaired epithelial tight junctions and increased intestinal permeability (35). In the present study, the MBF group exhibited increased serum IL-6 concentrations compared with healthy controls, whereas IL-6 levels in the FBF group were comparable to those in healthy animals. The differential response of individual cytokines after LPS stimulation may reflect distinct temporal dynamics during inflammatory activation and recovery (36, 37). Therefore, the observed IL-6 pattern may suggest that FBF supports recovery toward inflammatory homeostasis rather than exerting a broad suppressive effect on inflammatory mediators. Consistent with this interpretation, histological analysis showed improved epithelial organization and reduced inflammatory infiltration in FBF-treated mice, although quantitative scores did not reach statistical significance. These findings may reflect biologically relevant improvements that require validation in larger sample sizes or more sensitive molecular assessments.

Emerging evidence indicates that diet-induced modulation of gut microbiota represents an important mechanism linking nutrition with immune regulation (38, 39). In the present study, LPS exposure resulted in reduced microbial richness and altered community composition, consistent with previous observations in inflammatory states. FBF supplementation increased alpha diversity and shifted beta diversity toward a profile closer to healthy controls, suggesting improved ecological resilience following inflammatory disturbance. Taxonomic analyses further identified microbial signatures potentially associated with these beneficial benefits. *Erysipelotrichaceae* abundance increased in the MBF group but remained lower following FBF intervention. Although members of this family participate in carbohydrate breakdown and metabolism (40), enrichment of *Erysipelotrichaceae* has also been associated with inflammatory conditions in previous studies (41–43). In contrast, *Lachnospiraceae_NK4A136_group*, which has been linked to butyrate production (44), maintenance of epithelial barrier integrity, and reduced intestinal permeability (45, 46), was relatively preserved in FBF-treated mice. The functional relevance of such microbial shifts is substantiated by comparative evidence from dairy matrix studies. Sheep milk, but not cow milk, was shown to protect against DSS-induced colitis through selective restoration of the *Akkermansia*-SCFA-barrier axis (47). These findings highlight that dietary matrix composition, rather than individual nutrients alone, may critically determines microbiota-mediated host responses (47). However, because microbial metabolites were not directly measured in the present study, the functional significance of these microbial changes, particularly their relationship with SCFA production, remains to be experimentally validated.

The potential biological effects of FBF may be related to the integrated effects of whole food-derived components, dietary fiber, macronutrient composition, and other characteristics of the nutritional matrix. Unlike conventional milk-derived formulations, FBF incorporated diverse food-derived substrates including mixed grain powder, fruit and vegetable powder and prebiotics. Thus, it contained diverse types and substantially higher dietary fiber content as well as plant-derived carbohydrates. Such substrates may increase microbial fermentation (27), enhance the production of beneficial metabolites including SCFAs (48), and contribute to the regulation of inflammatory responses (23). This concept is consistent with the emerging framework of complex food matrices, in which multiple bioactive components interact synergistically to regulate physiological functions rather than acting independently (49). Although microbial metabolites were not measured in the current study, the observed enrichment of butyrate-associated taxa provide a rationale for future investigation into diet-microbiota-host interactions.

Importantly, our findings should be interpreted within the context of several limitations. First, the absence of an LPS-challenged basal-diet control group limited our conclusions to the comparison between FBF and MBF. Second, the single-dose LPS model may not fully reflect the complexity of inflammatory responses *in vivo*. Third, FBF and MBF were not nutrient-matched, and the specific contributions of individual nutrients (e.g., fiber, fat, energy intake) could not be fully determined as the formulas were provided ad libitum. Fourth, microbiota alterations were based on compositional analyses, and microbial metabolites (e.g., SCFAs), intestinal barrier markers, and inflammatory signaling pathways were not assessed precluding causal interpretation. Finally, the small sample size, use of male mice only, and potential cage effects may limit the generalizability of these findings. Future studies incorporating nutrient-matched controls, dietary intake monitoring, metabolomic analyses, barrier-related molecular assessments are needed to further elucidate the role of FBF in inflammatory recovery.

Despite these limitations, this study provides evidence that food-based nutritional strategies may exert biological effects extending beyond conventional nutritional supplementation. By influencing gut microbial composition and supporting recovery from inflammatory perturbation, FBF may represent a promising nutritional approach for supporting recovery in inflammation-associated conditions. Future research integrating metabolomic and mechanistic approaches are warranted to clarify how gut microbiota-host immune system interactions contribute to the effects of FBF (50, 51).

## 5 Conclusion

In conclusion, compared with MBF, FBF was associated with lower serum IL-6 levels following LPS challenge and induced distinct gut microbial compositional changes. FBF was also associated with alterations in microbial richness and diversity, including changes in the relative abundance of specific bacterial taxa such as *Erysipelotrichaceae* and *Lachnospiraceae_NK4A136_group*. These findings support FBF may promote recovery from low-grade inflammation. Further studies are needed to establish the underlying mechanisms and clinical relevance.

## Data Availability

The raw 16S rRNA sequencing data generated in this study have been deposited in the NCBI Sequence Read Archive (SRA) under BioProject accession number PRJNA1530810.

## Footnotes

1
https://cloud.oebiotech.com/
