# Supplementary material for: Food-based formula modulates gut microbial homeostasis and inflammatory responses following LPS challenge in mice
Source: Front Nutr. 2026 Oct 1;13:1932946. doi: 10.3389/fnut.2026.1932946 (PMC13668561; doi:10.3389/fnut.2026.1932946)
Supplement: Supplementary file 1 [file Supplementary_file_1.DOCX]

Table 1S. Composition and Formulation Design of the Food-based formula

| **Component** | **Source** | **Formulation Design** |
| --- | --- | --- |
| **Carbohydrate** | Cereal: Enzymatically hydrolyzed rice flour, gelatinized rice flour Tuber: Chinese yam powder | Cereals and tubers constitute >50% carbohydrate |
| **Fat** | Corn oil, Low-erucic-acid rapeseed oil (Canola oil), Coconut oil | - |
| **Protein** | Dairy-derived protein: Whey protein, Casein Plant-derived protein: Soy protein, Rice protein | Dairy: Plant protein = 2:1 |
| **Fiber** | Soluble: Galacto-oligosaccharides (GOS), Fructo-oligosaccharides (FOS) Insoluble: From natural ingredient sources(Vegetables: Broccoli powder, Tomato powder, Pumpkin powder, Kale powder, Carrot powder; Fruits: Apple powder, Strawberry powder) | Soluble: Insoluble = 4:1  GOS: FOS = 9:1;  Fruit and vegetable powders constitute 7–10% of the total formulation |

Table 2S. Histological scoring criteria.

| Items | Scoring standard | Scores |
| --- | --- | --- |
| Crypt damage | None 0-10% 10-20% >20% | 0 1 2 3 |
| Inflammation infiltration | none  mild moderate severe | 0 1 2 3 |
| Goblet cell injury | none  mild moderate severe | 0 1 2 3 |
| Edema | absent present | 0 1 |

Figure 1S. Rarefaction curves of Observed Species (A) and Shannon (B).

(A) Rarefaction curves for observed ASVs. (B) Rarefaction curves for the Shannon index (ASV level).
